# Using artificial intelligence for exercise prescription in personalised health promotion: A critical evaluation of OpenAI’s GPT-4 model

**DOI:** 10.5114/biolsport.2024.133661

**Published:** 2023-12-13

**Authors:** Ismail Dergaa, Helmi Ben Saad, Abdelfatteh El Omri, Jordan M. Glenn, Cain C. T. Clark, Jad Adrian Washif, Noomen Guelmami, Omar Hammouda, Ramzi A. Al-Horani, Luis Felipe Reynoso-Sánchez, Mohamed Romdhani, Laisa Liane Paineiras-Domingos, Rodrigo L. Vancini, Morteza Taheri, Leonardo Jose Mataruna-Dos-Santos, Khaled Trabelsi, Hamdi Chtourou, Makram Zghibi, Özgür Eken, Sarya Swed, Mohamed Ben Aissa, Hossam H. Shawki, Hesham R. El-Seedi, Iñigo Mujika, Stephen Seiler, Piotr Zmijewski, David B. Pyne, Beat Knechtle, Irfan M Asif, Jonathan A Drezner, Øyvind Sandbakk, Karim Chamari

**Affiliations:** 1 Primary Health Care Corporation (PHCC), Doha, Qatar; 2 Research Laboratory Education, Motricité, Sport et Santé (EM2S) LR19JS01, High Institute of Sport and Physical Education of Sfax, University of Sfax, Sfax 3000, Tunisia; 3 High Institute of Sport and Physical Education of Kef, Jendouba, Kef, Tunisia; 4 University of Sousse, Farhat HACHED hospital, Research Laboratory LR12SP09 «Heart Failure», Sousse, Tunisia; 5 University of Sousse, Faculty of Medicine of Sousse, laboratory of Physiology, Sousse, Tunisia; 6 Surgical Research Section, Department of Surgery, Hamad Medical Corporation, Doha 3050, Qatar; 7 Neurotrack Technologies, Redwood City CA, USA; 8 College of Life Sciences, Birmingham City University, Birmingham, B15 3TN, UK; 9 Institute for Health and Wellbeing, Coventry University, Coventry, CV1 5FB, UK; 10 Sports Performance Division, National Sports Institute of Malaysia, Kuala Lumpur, Malaysia; 11 Postgraduate School of Public Health, Department of Health Sciences (DISSAL), University of Genoa, Genoa, Italy; 12 Interdisciplinary Laboratory in Neurosciences, Physiology and Psychology: Physical Activity, Health and Learning (LINP2), UFR STAPS (Faculty of Sport Sciences), UPL, Paris Nanterre University, Nanterre, France; 13 Research Laboratory, Molecular Bases of Human Pathology, LR19ES13, Faculty of Medicine, University of Sfax, Tunisia; 14 Department of Exercise science, Yarmouk University, Irbid, Jordan; 15 Department of Social Sciences and Humanities, Autonomous University of Occident, Los Mochis, Mexico; 16 Departamento de Fisioterapia, Instituto Multidisciplinar de Reabilitação e Saúde, Universidade Federal da Bahia, Brazil; 17 Centro de Educação Física e Desportos, Universidade Federal do Espírito Santo, Vitória, Espírito Santo, Brazil; 18 Department of Motor Behavior, Faculty of Sport Sciences, University of Tehran, Tehran, Iran; 19 Department of Creative Industries, Faculty of Communication, Arts and Sciences, Canadian University of Dubai, Dubai, United Arab Emirates; 20 Department of Physical Education and Sport Teaching, Inonu University, Malatya 44000, Turkey; 21 University of Aleppo Faculty of Medicine: Aleppo, Aleppo Governorate, Syria; 22 Department of Comparative and Experimental Medicine, Nagoya City University Graduate School of Medical Sciences, Nagoya 467-8601, Japan; 23 Department of Chemistry, Faculty of Science, Islamic University of Madinah, Madinah, 42351, Saudi Arabia; 24 International Research Center for Food Nutrition and Safety, Jiangsu University, Zhenjiang 212013, China; 25 International Research Center for Food Nutrition and Safety, Jiangsu University, Zhenjiang 212013, China; 26 Department of Physiology, Faculty of Medicine and Nursing, University of the Basque Country, Leioa, Basque Country; 27 Exercise Science Laboratory, School of Kinesiology, Faculty of Medicine, Universidad Finis Terrae, Santiago, Chile; 28 Department of Sport Science and Physical Education, University of Agder, Kristiansand, Norway; 29 Jozef Pilsudski University of Physical Education in Warsaw, Warsaw, Poland; 30 Research Institute for Sport and Exercise, University of Canberra, Canberra, ACT, Australia; 31 Institute of Primary Care, University of Zurich, Zurich, Switzerland; 32 Department of Family and Community Medicine, University of Alabama at Birmingham, Birmingham, Alabama, USA; 33 Center for Sports Cardiology, University of Washington, Seattle, Washington, USA; 34 Center for Elite Sports Research, Department of Neuromedicine and Movement Science, Norwegian University of Science and Technology, Trondheim, Norway; 35 Higher institute of Sport and Physical Education, ISSEP Ksar Saïd, Manouba University, Tunisia

**Keywords:** AI Challenges, AI Evaluation, Chatbot, ChatGPT, Digital Health, Exercise Optimization, Fitness Algorithms, Machine Learning, Personalized Medicine, Real-time Monitoring

## Abstract

The rise of artificial intelligence (AI) applications in healthcare provides new possibilities for personalized health management. AI-based fitness applications are becoming more common, facilitating the opportunity for individualised exercise prescription. However, the use of AI carries the risk of inadequate expert supervision, and the efficacy and validity of such applications have not been thoroughly investigated, particularly in the context of diverse health conditions. The aim of the study was to critically assess the efficacy of exercise prescriptions generated by OpenAI’s Generative Pre-Trained Transformer 4 (GPT-4) model for five example patient profiles with diverse health conditions and fitness goals. Our focus was to assess the model’s ability to generate exercise prescriptions based on a singular, initial interaction, akin to a typical user experience. The evaluation was conducted by leading experts in the field of exercise prescription. Five distinct scenarios were formulated, each representing a hypothetical individual with a specific health condition and fitness objective. Upon receiving details of each individual, the GPT-4 model was tasked with generating a 30-day exercise program. These AI-derived exercise programs were subsequently subjected to a thorough evaluation by experts in exercise prescription. The evaluation encompassed adherence to established principles of frequency, intensity, time, and exercise type; integration of perceived exertion levels; consideration for medication intake and the respective medical condition; and the extent of program individualization tailored to each hypothetical profile. The AI model could create general safety-conscious exercise programs for various scenarios. However, the AI-generated exercise prescriptions lacked precision in addressing individual health conditions and goals, often prioritizing excessive safety over the effectiveness of training. The AI-based approach aimed to ensure patient improvement through gradual increases in training load and intensity, but the model’s potential to fine-tune its recommendations through ongoing interaction was not fully satisfying. AI technologies, in their current state, can serve as supplemental tools in exercise prescription, particularly in enhancing accessibility for individuals unable to access, often costly, professional advice. However, AI technologies are not yet recommended as a substitute for personalized, progressive, and health condition-specific prescriptions provided by healthcare and fitness professionals. Further research is needed to explore more interactive use of AI models and integration of real-time physiological feedback.

## INTRODUCTION

The growth of artificial intelligence (AI) has generated countless possibilities in various fields, including healthcare and fitness [1–4]. With the ability to process vast amounts of data and generate personalised recommendations, AI has emerged as a captivating and promising tool in exercise prescription [1]. However, despite the rapid development and implementation of AI-driven fitness applications, the ability of these technologies to deliver personalised, effective and, most importantly, safe exercise regimens for individuals with specific health conditions involving varying severity or co-morbidities remains largely unexplored. Adherence to physical activity (PA) guidelines is indisputably a key determinant of health, with a profound impact on the prevention and management of a range of health conditions, including cardiovascular diseases [5], respiratory diseases [6], diabetes mellitus [7], mental health disorders (eg; anxiety [8, 9], depression [10]), and Alzheimer disease [11, 12]. However, exercise prescription is not a one-size-fits-all approach or solution [13]. Numerous factors, such as individual health status, age, lifestyle, and personal fitness goals, influence the effectiveness and safety of an exercise regimen, thereby necessitating personalised exercise programs [13]. While a multitude of fitness applications aiming to offer a degree of individualisation in exercise prescription have entered the market [14, 15], they often fail to encompass the complexity of specific health conditions and the influence of medications [16], and an individual’s psychosocial factors [17]. Accordingly, this scenario represents a notable limitation of these applications in their current form, potentially restricting their safe usability for individuals with specific health concerns.

The emergence of AI chatbots shows promise in filling the need for more automated exercise prescriptions. By integrating large-scale data analytics with machine learning algorithms, these AI-powered systems can generate personalised recommendations in various fields, including nutrition [18], mental health [19, 20], and fitness [4, 21]. Nevertheless, these technologies also harbour their own set of limitations [22–24], such as the lack of empathetic human touch, privacy concerns, and challenges in transforming broad guidelines into individualised recommendations.

The aim of the study was to critically assess the efficacy of exercise prescriptions generated by OpenAI’s Generative Pre-Trained Transformer 4 (GPT-4) model. These prescriptions, tailored for five hypothetical individuals with diverse health conditions and fitness goals, were subjected to evaluation by several leading experts in exercise prescription.

## MATERIALS AND METHODS

This research was a simulation study designed to assess the potential of OpenAI’s GPT-4 model in producing individualised exercise programs for hypothetical patients with diverse health conditions and fitness goals. The study comprised four main phases: i) Scenario Creation, ii) GPT-4 prompt, iii) Exercise program generation (presented in the results section) and iv) Expert evaluation (presented in the Discussion section).

### Scenario Creation

**FIG. 1:**
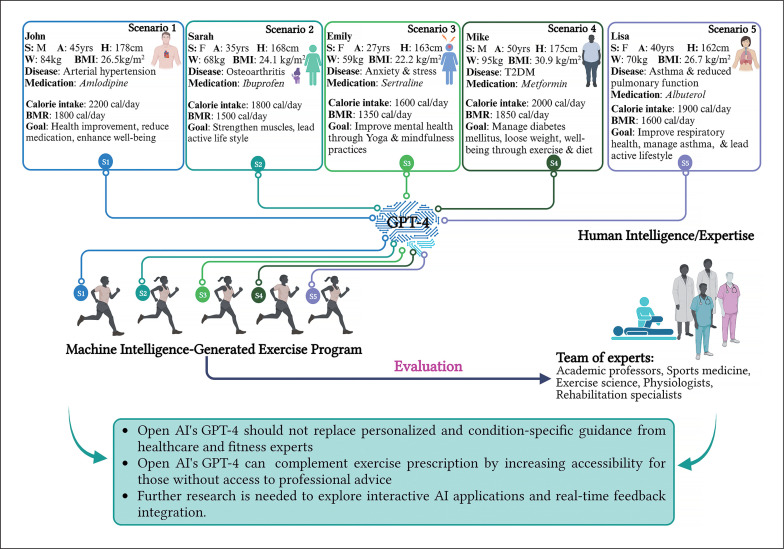
Graphical Abstract AI-sports

Five hypothetical patient profiles were created by the principal investigators (ID, HBS, KC in the authors’ list) with formal training and expertise in sports medicine, exercise science, and exercise physiology (Figure 1). The profiles included variables such as sex, age, height, weight, body mass index (BMI), medical conditions, medications intake, mean calorie intake, basal metabolic rate (BMR, estimated using the Harris-Benedict equations for females [25] and males [26]), motivations (ie; specific fitness goals), level of PA, and profession or occupation. The scenarios were intentionally diverse to represent a wide range of health conditions and fitness objectives prevalent in the general population.

### GPT-4 Prompt

We tasked GPT-4 with crafting a consistent fitness program suited for five distinct scenarios. The unified directive was as follows: *“Create a well-rounded 30-day fitness program table that seamlessly integrates workout sessions and rest days. To optimize the effectiveness of this routine, pay special attention to the FITT concept, which dictates the Frequency (how often), Intensity (how hard), Time (how long), and Type (what kind) of exercises to incorporate. Use the rate of perceived exertion (RPE) scale, a measure of how intense the exercise feels, to tailor the program to individual comfort levels. For the cardiovascular exercises, apply heart rate (HR) monitoring, a reliable method to adjust the exercise intensity, ensuring it is just right for each individual. For the strength-building exercises, adopt the one repetition maximum (1RM) principle, a standard that indicates the maximum weight someone can lift for one repetition, to set the appropriate workout intensity.”*

### Exercise Program Generation and Evaluation

Upon generating the programs, a comprehensive evaluation was undertaken by a panel of experts in exercise prescription. This distinguished panel comprised academic professors and doctors from the fields of sports medicine, exercise science, clinical exercise physiology, and rehabilitation.

The evaluators were from a diverse set of twenty countries, including: Australia, the Basque Country, Brazil, Chile, China, France, Iran, Italy, Japan, Jordan, Malaysia, Mexico, Norway, Poland, Qatar, Syria, Switzerland, Tunisia, the United Kingdom, the United Arab Emirates, and the United States of America. This broad geographic representation ensured a reflection of varied national norms and practices in exercise prescription. Each evaluator held a Ph.D. in exercise science and/or medicine and occupied a significant role in academia, with many serving as editors, associate editors or editor-in-chief for one or more esteemed academic journals in the field.

To mitigate subjective judgments and enhance the study’s objectivity, we intentionally selected evaluators from different countries, backgrounds, and educational experiences. We aimed to minimize subjectivity, ensuring a comprehensive and accurate evaluation by consolidating feedback from all contributors. Furthermore, to bolster the study’s reliability, we followed the criteria of “Defining the Role of Authors and Contributors” as outlined by the International Committee of Medical Journal Editors guidelines [27]. Only those who made substantial contributions to the study were listed as authors. Contributors who made less significant contributions or improvements to the manuscript were duly acknowledged.

In the methodology of our research, we adopted an innovative approach consistent with the study’s pioneering spirit. While conventional methodologies favour traditional evaluation scales, we intentionally chose a quasi-qualitative direction. This decision was made to benefit from the collective expertise and insights of our international panel of experts in exercise prescription. Rather than restricting ourselves to a quantitative scale, our methodology leaned towards this richer approach, aptly aligning with the unique scope of our study. It is pertinent to note a potential limitation here: the absence of a traditional scale, which some might anticipate in evaluations of this nature. Under the supervision of lead investigators (ID, HBS, and KC in the authors’ list), our evaluation strategy was subjected to a rigorous three-stage refinement process. The initial phase involved sharing a draft with co-authors for feedback. This was followed by stages of iterative refinement based on collective insights, and the process concluded with a final review for accuracy and completeness. For brevity, certain GPT-4 outputs were reformatted into 10 tables without any other modification.

### Single-Prompt Approach: Our Methodology Behind AI Interaction

In the process of generating exercise programs using the GPT-4 model, our approach was designed to mirror a typical patient experience with ChatGPT. The prompts provided to the AI model contained basic information that any sports enthusiast or beginner might readily find on the internet. This methodology was chosen to simulate a real-world scenario where individuals might seek exercise advice without the benefit of multiple rounds of expert interaction.

As sports scientists and exercise medicine specialists, we recognize that iterative interactions with the model, refining and adjusting inputs, could potentially yield a more precise and tailored exercise program. Such a program would inevitably reflect the unique background, expertise, and preferences of a practicing sports scientist. However, two primary reasons guided our decision against this approach: i) Iterative interactions were not the primary objective of our study. Our focus was on assessing the model’s capability to generate exercise prescriptions based on a singular, initial interaction, akin to a typical, lay person user experience; and ii) the scope and length of our study, already extensive (with over 7700 words and 10 tables), limited the feasibility of conducting multiple rounds of interactions and evaluations for each scenario. Engaging in such a detailed iterative process would have been feasible for a single case report, but not for the five diverse scenarios we chose to present in this study. For clarity and precision, it is vital to note that the creation of the scenarios and the responses from GPT-4 occurred on 25 June 2023. Given that ChatGPT’s performance can evolve over time, underscoring this specific date ensures the context and accuracy of our study are well-documented.

## RESULTS

### Scenario 1

#### A. Author Prompt

John – Cardiovascular Health Focus

– Sex: Male
– Age: 45 years
– Height: 178 cm
– Weight: 84 kg
– BMI: 26.5 kg/m^2^
– Medical Condition: Arterial hypertension
– Medications: John is currently taking *Amlodipine*, a calcium channel blocker, to manage his blood pressure.
– Mean calorie intake: 2,200 calories per day
– BMR: Approximately 1,800 calories per day
– Motivation: Improve cardiovascular health, reduce reliance on medication, and enhance overall well-being through regular PA.

#### B. GPT-4 response

Given John’s hypertensive condition and goals, a combination of cardio, strength, and flexibility training would be beneficial. Cardio exercise helps lower blood pressure, strength training can aid in weight management, and flexibility exercises improve overall body functions. All exercises should be performed at a moderate intensity (RPE 4–6 out of 10). Here is a sample 30-day exercise program (Table 1). This program alternates between cardio- and strength-training days, with one day of rest every third day. The “strength training (whole body)” refers to a mix of exercises that target all major muscle groups. For example, it could include squats, lunges, push-ups, seated rowing, and core exercises. As always, John should consult with his doctor before starting this or any other exercise program. He should also monitor his blood pressure regularly and pay attention to how he feels during exercise, especially if he experiences any discomfort or unusual symptoms.

**TABLE 1:** Scenario 1: John’s 30-day exercise program generated by generative pre-trained transformer 4.

Day	Exercise	Duration (min)	Intensity
1	Walking	30	Moderate (60–70% MHR)

2	Strength Training (Whole body)	30	Moderate (60–75% 1RM)

3	Rest day		

4	Cycling	30	Moderate (60–70% MHR)

5	Strength Training (Whole body)	30	Moderate (60–75% 1RM)

6	Rest day		

7	Walking	30	Moderate (60–70% MHR)

8	Strength Training (Whole body)	30	Moderate (60–75% 1RM)

9	Rest day		

10	Cycling	30	Moderate (60–70% MHR)

11	Strength Training (Whole body)	30	Moderate (60–75% 1RM)

12	Rest day		

13	Walking	30	Moderate (60–70% MHR)

14	Strength Training (Whole body)	30	Moderate (60–75% 1RM)

15	Rest day		

16	Cycling	30	Moderate (60–70% MHR)

17	Strength Training (Whole body)	30	Moderate (60–75% 1RM)

18	Rest day		

19	Walking	30	Moderate (60–70% MHR)

20	Strength Training (Whole body)	30	Moderate (60–75% 1RM)

21	Rest day		

22	Cycling	30	Moderate (60–70% MHR)

23	Strength Training (Whole body)	30	Moderate (60–75% 1RM)

24	Rest day		

25	Walking	30	Moderate (60–70% MHR)

26	Strength Training (Whole body)	30	Moderate (60–75% 1RM)

27	Rest day		

28	Cycling	30	Moderate (60–70% MHR)

29	Strength Training (Whole body)	30	Moderate (60–75% 1RM)

30	Rest day		

1RM: One repetition maximum. MHR: Maximal heart rate.

#### C. Interaction with GPT-4

##### C.1. Author Prompt

Could you consider elaborating on the workout sessions, perhaps by detailing changes in intensity within a single session? Also, it might be beneficial to incorporate the RPE scale as an additional measure of exercise intensity.

##### C.2. GPT-4’s response

Absolutely, I can definitely provide a more detailed session breakdown. I will provide an example for a walking session and a strength training session (Table 2).

**TABLE 2:** Scenario 1: Interaction with generative pre-trained transformer 4 (GPT-4).

Detailed walking session generated by GPT-4			

Time (min)	Activity	Intensity	Rate of perceived exertion
0–5	Warm-up: slow walking	Low (50% MHR)	2 to 3

5–10	Walking	Moderate (60% MHR)	4 to 5

10–20	Walking	Moderate-High (70% MHR)	6 to 7

20–25	Walking	Moderate (60% MHR)	4 to 5

25–30	Cool-down: slow walking	Low (50% MHR)	2 to 3

**Detailed strength training session (whole body) generated by GPT-4**			

**Time (min)**	**Exercise**	**Sets × Reps (second); Intensity**	**Rate of perceived exertion**

0–5	Warm-up: light cardio, dynamic stretching	Not applied; Low	2 to 3

5–10	Squats	3 × 10; Moderate (60% 1RM)	5 to 6

10–15	Lunges	3 × 10; Moderate (60% 1RM)	5 to 6

15–20	Push-ups	3 × 10; Moderate (60% 1RM)	5 to 6

20–25	Seated Row	3 × 10; Moderate (60% 1RM)	5 to 6

25–30	Core exercises (eg; planks)	3 × 30; Moderate	5 to 6

30–35	Cool-down: static stretching	Not applied; Low	2 to 3

1RM: One repetition maximum. MHR: Maximal heart rate.

### Scenario 2

#### A. Author Prompt

Sarah – Musculoskeletal Strength Emphasis

– Sex: Female
– Age: 35 years
– Height: 168 cm
– Weight: 68 kg
– BMI: 24.1 kg/m²
– Medical condition: Osteoarthritis
– Medications: Sarah occasionally takes over-the-counter anti-inflammatory medication, like *Ibuprofen*, to alleviate joint discomfort.
– Mean calorie intake: 1,800 calories per day
– BMR: Approximately 1,500 calories per day
– Motivation: Strengthen muscles to support joints, overcome physical challenges, and lead a more active lifestyle.

#### B. GPT-4’s response

Given Sarah’s osteoarthritis condition, a combination of strength training, low-impact cardio, and flexibility exercises would be beneficial. Strength training can help strengthen the muscles surrounding her joints, which can alleviate some of the pressure and reduce pain. Low-impact cardio exercises are easier on the joints while helping improve cardiovascular health. Flexibility exercises can help maintain joint mobility and function. Here is a sample 30-day exercise program, including more detailed session breakdowns for strength and cardio sessions (Table 3). “Strength Training (Whole body)” refers to a mix of exercises that target all major muscle groups, focusing on controlled movements to avoid joint strain. For example, it could include leg press, seated row, chest press, lat pull-down, and core exercises.

**TABLE 3:** Scenario 2: Sarah’s 30-day exercise program generated by generative pre-trained transformer 4.

Day	Exercise	Duration (min)	Intensity
1	Strength Training (Whole body)	45	Moderate (60–75% 1RM)

2	Rest day		

3	Low-impact Cardio (Swimming)	30	Moderate (60–70% MHR)

4	Rest day		

5	Strength Training (Whole body)	45	Moderate (60–75% 1RM)

6	Rest day		

7	Low-impact Cardio (Swimming)	30	Moderate (60–70% MHR)

8	Rest day		

9	Strength Training (Whole body)	45	Moderate (60–75% 1RM)

10	Rest day		

11	Low-impact Cardio (Swimming)	30	Moderate (60–70% MHR)

12	Rest day		

13	Strength Training (Whole body)	45	Moderate (60–75% 1RM)

14	Rest day		

15	Low-impact Cardio (Swimming)	30	Moderate (60–70% MHR)

16	Rest day		

17	Strength Training (Whole body)	45	Moderate (60–75% 1RM)

18	Rest day		

19	Low-impact Cardio (Swimming)	30	Moderate (60–70% MHR)

20	Rest day		

21	Strength Training (Whole body)	45	Moderate (60–75% 1RM)

22	Rest day		

23	Low-impact Cardio (Swimming)	30	Moderate (60–70% MHR)

24	Rest day		

25	Strength Training (Whole body)	45	Moderate (60–75% 1RM)

26	Rest day		

27	Low-impact Cardio (Swimming)	30	Moderate (60–70% MHR)

28	Rest day		

29	Strength Training (Whole body)	45	Moderate (60–75% 1RM)

30	Rest day		

1RM: One repetition maximum. MHR: Maximal heart rate.

For strength training and swimming (Table 4), let’s use a similar structure to the previous detailed sessions but adapted for Sarah. Sarah should adjust the intensity of her exercises based on her own perceived exertion, comfort level, and any joint pain. As always, she should consult with her doctor before starting this or any other exercise program, especially due to her osteoarthritis condition.

**TABLE 4:** Scenario 2: Interaction with generative pre-trained transformer 4 (GPT-4).

Detailed strength training session generated by GPT-4			

Time (min)	Exercise	Sets × Reps (seconds); Intensity	Rate of perceived exertion
0–5	Warm-up: light cardio, dynamic stretching	Not applied; Low	2 to 3

5–10	Leg Press	3 × 10; Moderate (60% 1RM)	5 to 6

10–15	Seated Row	3 × 10; Moderate (60% 1RM)	5 to 6

15–20	Chest Press	3 × 10; Moderate (60% 1RM)	5 to 6

20–25	Lat Pull-Down	3 × 10; Moderate (60% 1RM)	5 to 6

25–30	Core exercises (eg; planks)	3 × 30; Moderate	5 to 6

30–45	Cool-down: static stretching	Not applied; Low	2 to 3

**Detailed swimming session generated byGPT-4**			

**Time (min)**	**Activity**	**Intensity**	**Rate of perceived exertion**

0–5	Warm-up: slow swimming	Low (50% MHR)	2 to 3

5–10	Swimming	Moderate (60% MHR)	4 to 5

10–20	Swimming	Moderate-High (70% MHR)	6 to 7

20–25	Swimming	Moderate (60% MHR)	4 to 5

25–30	Cool-down: slow swimming	Low (50% MHR)	2 to 3

1RM: One repetition maximum. MHR: Maximal heart rate.

### Scenario 3

#### A. Author Prompt

Emily – Mental Health and Flexibility Target

– Sex: Female
– Age: 27 years
– Height: 163 cm
– Weight: 59 kg
– BMI: 22.2 kg/m²
– Medical condition: Anxiety and stress-related issues
– Medications: Emily is under the care of a psychiatrist and is prescribed Sertraline, an antidepressant, to manage her anxiety.
– Mean calorie intake: 1,600 calories per day
– BMR: Approximately 1,350 calories per day
– Motivation: Improve mental health, find inner peace, and achieve emotional balance through yoga and mindfulness practices.

#### B. GPT-4’s response

Given Emily’s goal of improving mental health and increasing flexibility, incorporating practices such as yoga, meditation, and low-intensity cardio exercise such as walking, or cycling can be highly beneficial. Yoga has been shown to improve flexibility, balance, and strength, while also having positive effects on mental health, reducing anxiety, and stress. Here is a sample 30-day exercise program, including more detailed session breakdowns for yoga and cardio sessions (Table 5).

**TABLE 5:** Scenario 3: Emily’s 30-day exercise program generated by generative pre-trained transformer 4.

Day	Exercise	Duration (min)	Intensity
1	Yoga	45	Moderate

2	Low-impact Cardio (Walking/Cycling)	30	Moderate (60–70% MHR)

3	Yoga	45	Moderate

4	Rest day		

5	Yoga	45	Moderate

6	Low-impact Cardio (Walking/Cycling)	30	Moderate (60–70% MHR)

7	Yoga	45	Moderate

8	Rest day		

9	Yoga	45	Moderate

10	Low-impact Cardio (Walking/Cycling)	30	Moderate (60–70% MHR)

11	Yoga	45	Moderate

12	Rest day		

13	Yoga	45	Moderate

14	Low-impact Cardio (Walking/Cycling)	30	Moderate (60–70% MHR)

15	Yoga	45	Moderate

16	Rest day		

17	Yoga	45	Moderate

18	Low-impact Cardio (Walking/Cycling)	30	Moderate (60–70% MHR)

19	Yoga	45	Moderate

20	Rest day		

21	Yoga	45	Moderate

22	Low-impact Cardio (Walking/Cycling)	30	Moderate (60–70% MHR)

23	Yoga	45	Moderate

24	Rest day		

25	Yoga	45	Moderate

26	Low-impact Cardio (Walking/Cycling)	30	Moderate (60–70% MHR)

27	Yoga	45	Moderate

28	Rest day		

29	Yoga	45	Moderate

30	Low-impact Cardio (Walking/Cycling)	30	Moderate (60–70% MHR)

MHR: Maximal heart rate.

For yoga and walking/cycling (Table 6), let’s use a similar structure to the previous detailed sessions but adapted for Emily. Emily should adjust the intensity of her exercises based on her own perceived exertion, comfort level, and any mental or physical discomfort. As always, she should consult with her doctor before starting this or any other exercise program, especially given her mental health condition.

**TABLE 6:** Scenario 3: Interaction with generative pre-trained transformer 4 (GPT-4).

Time (min)	Activity	Intensity	Rate of perceived exertion
**Detailed yoga session generated by GPT-4.**			

0–10	Warm-up: simple stretching and breathing exercises	Low	2 to 3

10–35	Yoga: sequences including a mix of standing, seated and supine postures	Moderate	5 to 6

35–45	Cool-down: relaxation and meditation	Low	2 to 3

**Detailed low-impact cardio session (walking/cycling) generated by GPT-4**			

0–5	Warm-up: slow walking/cycling	Low (50% MHR)	2 to 3

5–10	Walking/Cycling	Moderate (60% MHR)	4 to 5

10–20	Walking/Cycling	Moderate-High (70% MHR)	6 to 7

20–25	Walking/Cycling	Moderate (60% MHR)	4 to 5

25–30	Cool-down: slow walking/cycling	Low (50% MHR)	2 to 3

MHR: Maximal heart rate.

### Scenario 4

#### A. Author Prompt

Mike – Diabetes mellitus management and weight loss objective

– Sex: Male
– Age: 50 years
– Height: 175 cm
– Weight: 95 kg
– BMI: 30.9 kg/m²
– Medical Condition: Type 2 diabetes mellitus
– Medications: Mike takes *Metformin*, an oral diabetes medication, to manage his blood sugar levels.
– Mean calorie intake: 2,000 calories per day
– BMR: Approximately 1,850 calories per day
– Motivation: Manage diabetes effectively, lose weight to improve overall health, and enhance well-being through PA and proper nutrition.

#### B. GPT-4’s response

Given Mike’s objective of managing his diabetes mellitus and losing weight, a combination of regular cardio exercise, strength training, and dietary adjustments can be very beneficial. Regular PA can help improve insulin sensitivity, while a healthy diet can help manage blood glucose levels and contribute to weight loss. Here is a sample 30-day exercise program, including more detailed session breakdowns for strength and cardio sessions (Table 7). “Strength Training (Whole body)” refers to a mix of exercises that target all major muscle groups, focusing on controlled movements to avoid joint strain. For example, it could include leg press, seated row, chest press, lat pull-down, and core exercises.

**TABLE 7:** Scenario 4: Mike’s 30-day exercise program generated by generative pre-trained transformer 4.

Day	Exercise	Duration (min)	Intensity
1	Cardio (Walking/Running)	30	Moderate (60–70% MHR)

2	Strength Training (Whole body)	45	Moderate (60–75% 1RM)

3	Rest day		

4	Cardio (Walking/Running)	30	Moderate (60–70% MHR)

5	Strength Training (Whole body)	45	Moderate (60–75% 1RM)

6	Rest day		

7	Cardio (Walking/Running)	30	Moderate (60–70% MHR)

8	Strength Training (Whole body)	45	Moderate (60–75% 1RM)

9	Rest day		

10	Cardio (Walking/Running)	30	Moderate (60–70% MHR)

11	Strength Training (Whole body)	45	Moderate (60–75% 1RM)

12	Rest day		

13	Cardio (Walking/Running)	30	Moderate (60–70% MHR)

14	Strength Training (Whole body)	45	Moderate (60–75% 1RM)

15	Rest day		

16	Cardio (Walking/Running)	30	Moderate (60–70% MHR)

17	Strength Training (Whole body)	45	Moderate (60–75% 1RM)

18	Rest day		

19	Cardio (Walking/Running)	30	Moderate (60–70% MHR)

20	Strength Training (Whole body)	45	Moderate (60–75% 1RM)

21	Rest day		

22	Cardio (Walking/Running)	30	Moderate (60–70% MHR)

23	Strength Training (Whole body)	45	Moderate (60–75% 1RM)

24	Rest day		

25	Cardio (Walking/Running)	30	Moderate (60–70% MHR)

26	Strength Training (Whole body)	45	Moderate (60–75% 1RM)

27	Rest day		

28	Cardio (Walking/Running)	30	Moderate (60–70% MHR)

29	Strength Training (Whole body)	45	Moderate (60–75% 1RM)

30	Rest day		

1RM: One repetition maximum. MHR: Maximal heart rate.

For strength training and cardio sessions (Table 8), let’s use a similar structure to the previous detailed sessions but adapted for Mike.

**TABLE 8:** Scenario 4: Interaction with generative pre-trained transformer 4 (GPT-4).

Detailed strength training session generated by GPT-4			

Time (min)	Exercise	Sets × Reps (second); Intensity	Rate of perceived exertion
0–5	Warm-up: light cardio, dynamic stretching	Not applied; Low	2 to 3

5–10	Leg Press	3 × 10; Moderate (60% 1RM)	5 to 6

10–15	Seated Row	3 × 10; Moderate (60% 1RM)	5 to 6

15–20	Chest Press	3 × 10; Moderate (60% 1RM)	5 to 6

20–25	Lat Pull-Down	3 × 10; Moderate (60% 1RM)	5 to 6

25–30	Core exercises (eg; planks)	3 × 30; Moderate	5 to 6

30–45	Cool-down: static stretching	Not applied; Low	2 to 3

**Detailed cardio session (walking/running) generated GPT-4**			

**Time (min)**	**Activity**	**Intensity**	**Rate of perceived exertion**

0–5	Warm-up: slow walking	Low (50% MHR)	2 to 3

5–10	Walking/Running	Moderate (60% MHR)	4 to 5

10–20	Walking/Running	Moderate-High (70% MHR)	6 to 7

20–25	Walking/Running	Moderate (60% MHR)	4 to 5

25–30	Cool-down: slow walking	Low (50% MHR)	2 to 3

1RM: One repetition maximum. MHR: Maximal heart rate.

### Scenario 5

#### A. Author Prompt

Lisa – Respiratory Health and Pulmonary Function

– Sex: Female
– Age: 40 years
– Height: 162 cm
– Weight: 70 kg
– BMI: 26.7 kg/m²
– Medical condition: Asthma and reduced pulmonary function
– Medications: Lisa uses an inhaler (Albuterol) as a bronchodilator to manage her asthma symptoms.
– Mean calorie intake: 1,900 calories per day
– BMR: Approximately 1,600 calories per day
– Fitness objective: Improve respiratory health and lung capacity, manage asthma effectively, and lead an active lifestyle.

#### B. GPT-4’s response

Given Lisa’s objective of improving her respiratory health and pulmonary function, a combination of regular low-impact cardio and resistance exercises, along with breathing exercises, could be very beneficial. Regular PA can help increase lung capacity, improve cardiovascular health, and enhance overall fitness. Additionally, specific breathing exercises can help strengthen the respiratory muscles, increase lung function, and manage asthma symptoms effectively. Here is a sample 30-day exercise program for Lisa (Table 9). “Resistance Training (Whole body)” refers to a mix of exercises that target all major muscle groups, focusing on controlled movements and proper breathing techniques to avoid respiratory strain.

**TABLE 9:** Scenario 1: Lisa’s 30-day exercise program generated by generative pre-trained transformer 4.

Day	Exercise	Duration (min)	Intensity
1	Cardio (Swimming)	30	Moderate (60–70% MHR)

2	Breathing Exercises (Pursed-lip and Diaphragmatic Breathing)	15	Low

3	Resistance Training (Whole body)	45	Moderate (60–75% 1RM)

4	Rest day		

5	Cardio (Cycling)	30	Moderate (60–70% MHR)

6	Breathing Exercises (Pursed-lip and Diaphragmatic Breathing)	15	Low

7	Resistance Training (Whole body)	45	Moderate (60–75% 1RM)

8	Rest day		

9	Cardio (Swimming)	30	Moderate (60–70% MHR)

10	Breathing Exercises (Pursed-lip and Diaphragmatic Breathing)	15	Low

11	Resistance Training (Whole body)	45	Moderate (60–75% 1RM)

12	Rest day		

13	Cardio (Cycling)	30	Moderate (60–70% MHR)

14	Breathing Exercises (Pursed-lip and Diaphragmatic Breathing)	15	Low

15	Resistance Training (Whole body)	45	Moderate (60–75% 1RM)

16	Rest day		

17	Cardio (Swimming)	30	Moderate (60–70% MHR)

18	Breathing Exercises (Pursed-lip and Diaphragmatic Breathing)	15	Low

19	Resistance Training (Whole body)	45	Moderate (60–75% 1RM)

20	Rest day		

21	Cardio (Cycling)	30	Moderate (60–70% MHR)

22	Breathing Exercises (Pursed-lip and Diaphragmatic Breathing)	15	Low

23	Resistance Training (Whole body)	45	Moderate (60–75% 1RM)

24	Rest day		

25	Cardio (Swimming)	30	Moderate (60–70% MHR)

26	Breathing Exercises (Pursed-lip and Diaphragmatic Breathing)	15	Low

27	Resistance Training (Whole body)	45	Moderate (60–75% 1RM)

28	Rest day		

29	Cardio (Cycling)	30	Moderate (60–70% MHR)

30	Breathing Exercises (Pursed-lip and Diaphragmatic Breathing)	15	Low

1RM: One repetition maximum. MHR: Maximal heart rate.

Here is a detailed breakdown of Lisa’s resistance training session (Table 10). For each exercise, Lisa should take slow, deep breaths, exhaling on the exertion phase and inhaling on the return. If she feels any shortness of breath, she should slow down or take a break, and use her inhaler if necessary. Breathing exercises are also an integral part of her program. Pursed-lip breathing involves inhaling through the nose and exhaling slowly through puckered lips, while diaphragmatic breathing focuses on fully engaging the diaphragm, not just the chest, during breaths. These exercises can help increase lung capacity and improve respiratory muscle function, thereby helping to manage asthma symptoms. As always, she should consult with her doctor before starting this or any other exercise program, especially given her asthma condition.

**TABLE 10:** Scenario 5: Detailed resistance training session generated by generative pre-trained transformer 4.

Time (min)	Exercise	Sets × Reps (second); Intensity	Rate of perceived exertion
0–5	Warm-up: light cardio, dynamic stretching	Not applied; Low	2 to 3

5–10	Leg Press	3 × 10; Moderate (60% 1RM)	5 to 6

10–15	Seated Row	3 × 10; Moderate (60% 1RM)	5 to 6

15–20	Chest Press	3 × 10; Moderate (60% 1RM)	5 to 6

20–25	Lat Pull-Down	3 × 10; Moderate (60% 1RM)	5 to 6

25–30	Core exercises (eg; planks)	3 × 30; Moderate	5 to 6

30–45	Cool-down: static stretching	Not applied; Low	2 to 3

1RM: One repetition maximum.

## DISCUSSION

The primary objective of this study was to critically assess the efficacy of exercise prescriptions generated by OpenAI’s GPT-4 model for five hypothetical patient profiles with diverse health conditions and fitness goals. Our focus was on assessing the model’s capability to generate exercise prescriptions based on a singular, initial interaction, akin to a typical user experience. This evaluation was conducted by leading experts in the field of exercise prescription. Through this assessment, we aimed to understand the potential and limitations of AI, specifically GPT-4, in crafting personalised exercise programs, juxtaposing its outputs against the insights and expertise traditionally provided by human professionals in the domain.

### Evaluation and Review: Scenario 1 – John

John’s case represents a typical middle-aged individual suffering from arterial hypertension, aiming to enhance his cardiovascular health and reduce medication dependence through a structured exercise program. The generated 30-day regimen shows a commendable application of the FITT principles, incorporating a blend of cardiovascular exercises, resistance training, and flexibility routines, all of which are demonstrably beneficial for hypertensive individuals and cardiovascular health in general [28, 29].

The program’s heavy reliance on moderate-intensity workouts is in line with recommendations for hypertensive patients, reducing the likelihood of excessive spikes in blood pressure during exercise [30]. This approach aligns with a conservative strategy that prioritizes understanding how the individual reacts to moderate-intensity exercise before exploring more intense options.

While emerging research indicates that high-intensity interval training (HIIT) could offer substantial cardiovascular and hypertensive benefits [31, 32], the decision to exclude HIIT from the initial 30-day program is consistent with a personalized and cautious approach. A human practitioner might rationalize current decisions based on prior experiences, successes, and failures. For instance, if a practitioner had previously observed a patient with similar health conditions to John experiencing adverse reactions to HIIT, they might be more cautious and exclude HIIT from John’s program. In this context, the exclusion of HIIT seems prudent, reflecting the practitioner’s accumulated knowledge and experience over time. The ChatGPT-formulated program, while based on prevalent information from the Internet, might prioritise information that is more abundant or historically prevalent over newer, potentially more relevant expert insights. This means that while GPT-4 can access a vast amount of information, it may not always prioritise the most recent or expertendorsed data. As a result, traditional practices with a wealth of online information might be favoured over newer, updated methodologies. This highlights a limitation in the model’s ability to discern the quality and relevance of the information it accesses.

Introducing HIIT might be a valuable addition to the program in the future, but only after careful monitoring and assessment of the individual’s response to the initial regimen. This approach recognises that exercise prescription can be subjective and based on individual preferences and needs, and emphasises the importance of personalised care in managing hypertensive conditions. A prudent approach also acknowledges the nuanced judgment that human practitioners bring to exercise prescription, balancing evidence-based practice with individualized care.

While the HR-based approach for managing workout intensity is noteworthy, it would have been beneficial to incorporate specific HR zones for each cardio session [33]. For hypertensive patients like John, individualized HR zone training could prove particularly beneficial [34]. A common approach to establishing these zones involves first estimating the predicted maximal HR (MHR) (usually using the “220-age” formula [35]). Then, the Karvonen formula [36] [target training HR = resting HR + (0.6 × (MHR – resting HR))] is applied, followed by calculating different training zones as percentages of this target HR [37]. However, one should be cautious with the use of predicted MHR calculations as several estimation issues should not be overlooked; ideally, the real MHR of the individual should be measured by qualified practitioners [38]. Indeed, if a patient estimates his/her HR zones using an inappropriate formula, it could lead to an inadequate exercise intensity. GPT-4 did not mention any details about how to calculate those HR zones, and predicted MHR. This shortcoming might not only hinder the effectiveness of the exercise program but also pose potential health risks, particularly for individuals with underlying medical conditions such as arterial hypertension. Accurate calculation and understanding of HR zones are essential for tailoring an exercise regimen that is both safe and effective for the individual’s specific needs and goals.

Resistance training has sensibly been included in the program, aligning with research demonstrating its positive impact on blood pressure and cardiovascular health [39, 40]. Nonetheless, high-resistance strength training may lead to a significant transient increase in systolic and diastolic blood pressure [41]. Consequently, a program design leaning towards lower resistance with higher repetitions might be a safer alternative for John, while still providing the benefits of resistance training, without the associated acute blood pressure elevation [42].

An evident progression in terms of exercise duration is a strong point of the program. However, it appears to overlook the progression in other crucial areas, such as workout intensity, the weight used in resistance training, and the complexity of exercises. A program incorporating systematic progression in these elements might stimulate continual adaptation and improvement, offering greater benefits for John. Another element seemingly absent from the program is RPE monitoring [43]. RPE could provide a more reliable measure of workout intensity than HR alone [44].

The program effectively integrates rest days, underscoring their importance in facilitating recovery and adaptation. However, the current placement of these rest days, predominantly after strength training sessions, raises questions. Strategically positioning rest days after high-intensity workouts or after a series of consecutive moderate-intensity sessions can optimize muscle recovery and reduce the risk of overtraining [45]. Thus, re-evaluating the sequencing of rest days in this context may enhance the overall effectiveness of the program. Furthermore, it is essential to note the increase in self-paced exercise recommendations, especially for specific populations. Such exercises allow individuals to adjust their workout intensity based on their comfort and capability, which can be particularly beneficial for those taking certain medications that might affect their physiological response to exercise. The GPT-4 program also seemed limited by meeting (but not surpassing) the PA guideline of 150 minutes per week of moderate to vigorous PAs. The necessity of two rest days per week should also be questioned as some patients may prefer to exercise in smaller “doses” more than 5 times per week.

Additionally, the importance of isometric exercises and varied contraction regimens cannot be overlooked. Different health disorders may necessitate specific contraction patterns to maximize therapeutic benefits. A recent network meta-analysis reported that isometric exercise may provide the largest improvement in resting blood pressure [40]. For instance, isometric exercises, which involve muscle contractions without any significant movement, can be especially beneficial for individuals with certain joint or muscular disorders. The program could benefit from integrating these exercises, tailoring the contraction regimen to the specific needs of the individual’s health condition. However, patients should be warned to avoid the Valsalva manoeuvre while performing isometric exercises to avoid any overload on the cardiovascular system, and there were no cautions highlighted on this aspect by the proposed program [46].

Lastly, even if not prompted for, we believe that the proposed program should have touched on the patient diet with at least a list of food to avoid in association with arterial hypertension. Indeed, any clinical practitioner if confronted with suggesting an exercise-based program for hypertensive patient will most probably consider associated risk factors like nutrition or sleep.

The exercise program generated by GPT-4 exhibits a solid grasp of fundamental exercise prescription principles. However, there is room for refinement in areas like individualised intensity measures, systematic progression, nuanced application of resistance training guidelines, and incorporation of RPE as a training monitoring tool. With these adjustments, the program could potentially provide further health benefits for John.

### Evaluation and Review: Scenario 2 – Sarah

Sarah, a 35-year-old female diagnosed with osteoarthritis, aspires to augment her musculoskeletal strength, mitigate her joint discomfort, and live a more active lifestyle. The AI model’s proposed exercise plan, designed around FITT principles, provides a suitable starting point for such objectives. However, the program warrants a more detailed critique, particularly in the context of osteoarthritis management and the potential therapeutic benefits of PA.

First, the model appropriately prescribes strength-training exercises, a cornerstone of non-pharmacological management for osteoarthritis [47]. Not only does resistance training improve muscular strength, but enhances joint stability and function and alleviating osteoarthritis symptoms [47]. However, the prescription lacks specificity in terms of the intended load, repetitions, and sets for each exercise, which might influence the therapeutic effectiveness.

To illustrate, a typical exercise prescription for someone like Sarah might specify that she should perform leg presses at 60% of her 1RM, for three sets of 10 repetitions, with a 2-minute rest in-between. This level of detail ensures that the exercise is tailored to her current strength level, providing a clear progression path. However, there was no instruction for how she safely determine her 1RM for each exercise. The AI’s recommendation might have been more generic, indicating leg presses while specifying the weight, repetitions, and sets. Such a lack of specificity, while more general and perhaps easier to adopt, could however lead to suboptimal results and even potential injury if Sarah were to guess the appropriate weight or overexert herself.

Secondly, the AI model rightly includes flexibility exercises in the program, which are crucial for maintaining joint range of motion and reducing stiffness associated with osteoarthritis [48]. However, the model could have suggested exercises for enhancing proprioception and neuromuscular control exercises, such as balance and agility training, which are often compromised in individuals with osteoarthritis [49].

Regarding cardiovascular exercises, the model adequately introduces low-intensity activities (ie; walking). This aspect is critical considering the load-bearing nature of osteoarthritis, particularly as it affects Sarah’s lower limbs. Nevertheless, the model could have suggested non-weight-bearing activities such as cycling, which may reduce joint stress while promoting cardiovascular fitness [50].

Furthermore, the program could have been further individualised by considering the affected joints and tailoring the exercises accordingly. For example, if Sarah’s osteoarthritis affects her knees, certain exercises such as deep squats shall be avoided due to excessive knee joint load [51, 52]. It is worth noting that while there were traditional reservations about land-based exercises for individuals with osteoarthritis, recent research and clinical practices have evolved. Land-based exercises, when performed with proper technique and under expert guidance, can be beneficial for osteoarthritis patients [51, 52]. They can help improve joint mobility, muscle strength, and overall functional capacity. Moreover, these exercises can be adapted to ensure minimal joint stress while maximizing therapeutic benefits. For instance, shallow squats or partial range movements can be introduced to reduce undue strain on affected joints. The key lies in the correct execution, progression, and individualisation of these exercises to suit the specific needs and limitations of the osteoarthritis patient. This aspect is overlooked by the model.

The information provided to Sarah also recommended an evaluation by a doctor before commencing her exercise program. This recommendation, which is based on older guidelines, is incorrect since new guidelines support that low-risk patients do not require a screening examination or “medical clearance” to begin an exercise program [53]. Of course, Sarah could pursue this for added safety, but the GPT-4 model could have presented this option without making it a barrier for those with poor access to care.

Finally, the model logically incorporates rest days into the program, which are essential for promoting tissue healing and recovery, thus reducing the risk of exacerbating Sarah’s osteoarthritis symptoms. However, Sarah could engage in light activities such as stretching or yoga on “rest” days, thus promoting flexibility and relaxation, adding value without hampering recovery. A more detailed, tailored, and multifaceted approach that considers the specific nuances of her osteoarthritis could better cater to Sarah’s needs, and help her achieve her fitness objectives more effectively and safely.

### Evaluation and Review: Scenario 3 – Emily

Emily is a 27-year-old individual grappling with anxiety and stress-related issues, and managed with *Sertraline*, an antidepressant. Her objective was to enhance her mental health, find inner peace, and achieve emotional balance through yoga and mindfulness practices. Given the increasing popularity and frequent requests for yoga as a potential solution for such health conditions, the AI model suggests a detailed yoga-centric program, constructed in adherence to FITT principles. While Yoga is a commonly sought-after intervention, it is essential to acknowledge that many other behavioral and cognitive interventions have shown beneficial results. However, an in-depth analysis reveals some areas for potential improvement. Moreover, the program seems to overlook crucial factors like sleep quality and chronotype, which play a pivotal role in managing anxiety and stress.

The model appropriately emphasizes yoga and meditation, both of which are well-documented for their beneficial effects on mental health, stress reduction, and overall well-being [54]. The model correctly provides for a gradual increase in session length, permitting Emily to adapt progressively to the increasing demands of the program. However, the program could have been improved by providing specific details about the yoga poses and sequences suitable for stress relief, such as restorative or Yin Yoga, along with guidance on proper alignment and modification options to suit Emily’s comfort and skill level. This outcome may be possible through links to other resources and instructional videos. In addition, as with scenario 2, the GPT-4 model recommended a medical evaluation before beginning the exercise program which is not necessary in an otherwise healthy young adult.

The prescription of daily yoga sessions, coupled with mindfulness exercises, shows the model’s understanding of the importance of consistency in mental health-related physical activities. However, the prescription does not adequately account for the rest and recovery necessary to prevent potential physical and mental fatigue associated with daily yoga practices [55]. Incorporating rest days or days with lighter, restorative practices could potentially improve this aspect of the program.

The model’s program also includes a ‘perceived exertion’ measure, the Borg scale, to self-assess the intensity of the yoga sessions. While this can be a useful tool for cardio-based workouts, it may not be entirely suitable for yoga, where intensity can vary significantly based on the type of Yoga and individual postures [55]. A more appropriate measure might be a discomfort or difficulty scale, particularly suited to the slower pace and holding postures of yoga. Lastly, the model does not incorporate any form of aerobic or strength training exercises in Emily’s program [56, 57]. While her primary focus is on mental health and yoga practices, including these forms of exercises could provide complementary benefits. Aerobic exercises, for instance, are known to promote endorphin release, aiding in mood improvement [58], while strength training can boost self-esteem and body image, providing an additional mental health benefit [59].

### Evaluation and Review: Scenario 4 – Mike

Mike, a 50-year-old male diagnosed with type 2 diabetes mellitus, aims to manage his disease effectively, lose weight, and improve his overall health and well-being. The AI model recommends a program that combines cardiovascular, strength, and flexibility exercises, abiding by FITT principles. While this program aligns with general recommendations for managing diabetes mellitus and promoting weight loss, it lacks specificity and a more nuanced understanding of exercise prescription in the context of diabetes management.

The model correctly prescribes cardiovascular exercise as a vital component of Mike’s program, given its well-documented benefits in enhancing insulin sensitivity, aiding glucose control, and promoting weight loss [60]. However, it misses a crucial point: the potential rare risk of hypoglycemia during and following prolonged aerobic exercise for individuals on glucose-lowering medications [61, 62]. It is essential for patients like Mike, especially if they are on *metformin*, to be cognizant of exercise-induced glucose fluctuations. While *metformin* generally reduces the risk of exercise-induced hypoglycemia due to its mechanism of action on liver glucose production, some nuances need to be considered. A more personalised approach for *metformin* users would involve monitoring blood glucose levels pre- and post-exercise to understand any unexpected fluctuations. Individuals should also be made aware of the potential for gastrointestinal side effects of *metformin*, which might be exacerbated with exercise. As always, it is advisable to consult with a healthcare provider for individualised guidance, especially when initiating a new exercise regimen while on *metformin* [63, 64]. Furthermore, the inclusion of high-intensity low-volume endurance exercises, such as HIIT or moderate-intensity interval training, could offer additional benefits, and should be considered in the exercise prescription.

Strength training exercises are appropriately included, given their role in improving muscle mass and insulin sensitivity, thereby aiding in glucose control [65]. However, the program could be improved by specifying the types of resistance exercises to be performed, their order, and the amount of resistance (in relation to Mike’s actual or estimated 1RM), how to safely determine one’s 1RM, and facilitating the safe and effective execution of these exercises.

The program’s inclusion of flexibility exercises, which aid in maintaining joint health and overall mobility, is also commendable [48]. However, the model does not provide any specific stretching exercises or guidance on how these should be performed, which could be helpful for preventing potential injury [66].

Given that both hypoglycemia and hyperglycemia can occur in individuals with diabetes mellitus due to exercise, education about self-monitoring of blood glucose and guidance on appropriate responses to abnormal glucose levels are vital [67].

Furthermore, the model could have incorporated education about the signs of hypoglycemia and foot care in the exercise program, given that individuals with diabetes mellitus are often at increased risk of hypoglycemia and foot ulcers [68].

### Evaluation and Review: Scenario 5 – Lisa

Lisa, a 40-year-old female with asthma and impaired pulmonary function, has the objective of improving her respiratory health and lung capacity. The AI model proposes a program combining cardiovascular, strength, and flexibility exercises, framed within the FITT guidelines. The program captures a broad range of PAs beneficial for overall health; however, the specificity and customization to cater to Lisa’s specific condition seem to be lacking in certain aspects.

The model appropriately prescribes cardiovascular exercise, given its proven benefits in enhancing overall cardiorespiratory fitness [69]. However, the program could have been enhanced by incorporating exercises particularly known for their positive impacts on respiratory health [70]. Additionally, considering the benefits of eccentric exercises, which are less stressful on the cardiorespiratory system compared to other contraction regimens, their inclusion could be advantageous for Lisa. The inclusion of strength training is also pertinent, considering that improved muscle strength can help reduce the overall work of breathing and enhance Lisa’s exercise tolerance [71]. Still, a more targeted approach could involve additional exercises to specifically strengthen her respiratory muscles, such as incentive spirometry or breathing against resistance [72–74], beyond the pursed-lip and diaphragmatic breathing suggested by GPT-4.

The model’s program incorporates flexibility exercises, beneficial in maintaining overall joint and muscle health [75]. However, the program could be better tailored by suggesting exercises to help enhance thoracic mobility and lung function.

The model correctly uses RPE as a method for Lisa to gauge her exertion levels. However, given Lisa’s condition, additional guidance on recognising the signs of exercise-induced bronchoconstriction [76], such as unusual shortness of breath, chest tightness, or prolonged recovery, would have been beneficial.

Furthermore, the program does not address the importance of a proper warm-up and cool-down in preventing exercise-induced bronchoconstriction symptoms, an oversight that could negatively influence Lisa’s exercise experience and potentially increase the likelihood of drop out due to exercise related issues/discomfort. The model also did not review proper use of a rescue inhaler for symptom exacerbations during exercise, or the potential need for prophylactic use before exercise. However, the model did recommend that Lisa ensure her inhaler remains near-by, showing that the model considered her condition and the potential associated issues that the patient might encounter while exercising.

### Overall Evaluation and Interpretation

This study has explored the application of the GPT-4 model for personalised exercise prescription for five-example patient profiles with various health conditions. The GPT model’s responses were able to produce generally safe exercise programs grounded in the general FITT principles and RPE guidelines, demonstrating the potential utility of AI in generating basic exercise recommendations. However, the analysis revealed several limitations and areas for improvement.

The programs suggested by ChatGPT placed a significant emphasis on safety, often prescribing moderate-intensity exercises rather than high-intensity ones. While such a cautious approach is understandable, especially when introducing a new exercise regimen to individuals, it might be perceived as overly conservative. For a shortterm intervention over a month, this level of caution in the initial weeks might be excessive. Ideally, after establishing a baseline in the first couple of weeks, the intensity could be gradually increased. Starting from the third or fourth week, a small increase in intensity could better balance the goals of improving the patient’s health condition while still maintaining safety. However, this progression also depends on the level of fitness of the individual prior to the program (see more below). For an extremely unfit individual, a more prolonged preparation/transition period is warranted to diminish the likelihood of dropping out.

The model’s inability to monitor an individual’s physiological response and adjust in real-time is a limitation. While some emerging technologies are beginning to offer this capability, ChatGPT, as used here, cannot independently provide real-time feedback or adjustments. This caution restricts the potential for optimal progress and adaptation, which are essential for health improvement and the attainment of specific goals [77].

Furthermore, the program’s lack of preliminary patient assessment is a notable shortcoming. If the AI system were to request information such as the subject’s current physical condition, exercise experience, or the length of time suffering from the health condition, it could plan the exercise in a more individualised and effective manner. In practice, a human practitioner, with the ability to assess the individual’s responses in real-time, would likely provide a more varied, dynamic, and intense program, challenging the individual while ensuring safety and effectiveness with an appropriately individualized prescription [77].

The monotony in program design, highlighted in the present analysis, points towards the lack of variability, another crucial principle of training, in the GPT’s prescribed plans. While maintaining a certain level of consistency is necessary for habit formation and gradual adaptation, introducing regular changes in exercise modes, intensities, and volumes is essential to prevent plateaus in progress, enhance motivation, and foster long-term adherence [78].

It seems that unless a patient is extremely unfit, the exercise regimens developed by GPT-4 generally will allow individuals to maintain their current health status but fall short of providing the necessary stimuli for significant advancements in their health or fitness. Moreover, the holistic approach that leads to personalisation and creativity in exercise prescription goes beyond what AI-generated plans can currently offer. This approach considers unique user attributes and adapts in ways that resonate with their individual preferences and needs. Even with technological advances, the prospect of AI systems fully replacing human expertise in the exercise prescription field seems distant as of now (ie; August 2023).

Despite its shortcomings, the GPT model’s approach offers a costeffective alternative for individuals who cannot afford personal trainers, yet it falls short of replicating the personalised service and expertise that human trainers provide. While “digital humans” may provide a potential solution to facilitate knowledge transfer, motivation, and adherence, this study was designed only to evaluate the content and appropriateness of the exercise recommendations. Although not directly investigated in our study, the authors contend that the nuances of human interaction, motivation, and real-time adjustment of training plans based on various factors likely remain unparalleled by AI in its current state. As indicated in the methodology section, the decision to conduct this analysis without significant interaction with the GPT model was deliberate and guided by several considerations. Primarily, the study sought to assess ChatGPT’s ability to tailor training programs to specific patient requirements, simulating the experience of a typical untrained layperson lacking sports science knowledge. This approach allowed for critical evaluation of the program from the perspective of sports medicine and sports science specialists. Further iterative interactions with AI and text expansion were beyond the scope of this study. We also assert that a broader investigation of multiple health profiles would yield more valuable insights than a narrow focus on a single profile with numerous interactions. To ensure clarity, some evaluations, which are common between scenarios, were addressed in the overall evaluation to avoid redundancy. Despite these limitations, the study-maintained objectivity by adopting an observational approach, avoiding external factors that might alter the model’s responses. This method facilitated a clear differentiation between the model’s inherent knowledge and potential external influences.

Future research should explore the interactive capabilities of AI models, including ChatGPT, to determine if they can be harnessed to enhance the specificity and effectiveness of prescribed exercise programs. Such studies may also consider focusing on individual patient cases with multiple interactions to assess the model’s ability to generate better-tailored training programs over successive rounds of interaction. As AI technology advances, subsequent iterations of these models may overcome existing limitations, enabling patients and fitness professionals to utilize these tools for crafting more accessible, personalized, and effective exercise prescriptions. Such advancements can serve as valuable aids, amplifying the expertise of fitness professionals rather than replacing them. This evolution emphasizes the potential of AI to act as a collaborative tool, enriching the human element in the domain of fitness and health.

### Limitations

In evaluating the application of AI, specifically the GPT-4 model, for crafting personalized exercise programs, this study has uncovered both promising avenues and significant limitations. These constraints in addition to the limitations of our study must be carefully considered when interpreting the findings.

One primary limitation was the static nature of the interaction with the AI model. While personal and medical details were provided, along with a request for a 30-day exercise program, the model’s ability to refine and expand upon its initial recommendations through continued dialogue was not fully investigated. This approach led to somewhat generic recommendations, reflecting the study’s focus on a wide array of medical conditions rather than an in-depth exploration of a single case. Other studies should consider examining iterative interactions with AI throughout the course of an exercise program.

Our patient profiles were intentionally simplistic with a singular medical condition. Thus, our results may not be fully generalizable to the performance of AI in formulating an exercise program for patients with multiple co-morbidities or more severe medical disorders. This opportunity represents an important area of future research.

Another significant limitation is the quasi-qualitative aspect of the study. While this approach offers some advantages, especially given the pioneering nature of our research, the lack of a recognised and validated scale is a substantial concern for such evaluations. This gap underscores the necessity for future research dedicated to the development of validated scales for evaluating chatbots-generated programs, whether in sports medicine or broader medical science contexts.

Furthermore, the current technological limitations of AI, which preclude real-time physiological monitoring and responsive adjustments to exercise prescriptions, were evident in the model’s tendency to prescribe extremely conservative programs. Without real-time feedback, safety has been prioritised over challenge and progression, potentially hindering fitness and health improvements. This limitation is underscored by the contrast with other technological advancements, such as the “precision motion technology “offered by “Hinge Health” [79], which can provide the real-time monitoring currently beyond the reach of AI models like GPT-4.

The lack of specificity and customisation in addressing individual health conditions and goals also emerged as a limitation, impacting the effectiveness of the programs and possibly influencing users’ motivation and adherence. Additionally, the study’s omission of factors such as ethnicity, exercise preferences, profession, lifestyle habits, and other personal details further constrained the model’s ability to provide tailored fitness programs. Although attempts were made to incorporate these details, the model’s unresponsiveness led the authors to retain the original approach.

The model’s inclination towards safety over progression, while understandable given the lack of real-time feedback, could obstruct significant long-term fitness and health gains, especially for individuals with specific health conditions requiring more targeted programming. Finally, there are some individuals that will need the continuous presence of a coach next to them to exercise [80, 81], and for these persons AI may never provide an adequate solution.

Future research should pursue more dynamic and interactive designs, exploring continued dialogue with AI models, and integration of real-time physiological monitoring technology. Such advancements could enhance the specificity and effectiveness of AI-generated exercise programs, opening new possibilities for personalised fitness and health interventions.

## CONCLUSIONS

Our findings indicate that AI technology (in this case OpenAI’s GPT-4) can provide a baseline for safe exercise recommendations. However, the technology may not offer the degree of complexity and adaptability required for optimized long-term fitness and health improvement. The exercise regimens developed by GPT-4 permit individuals with average fitness to maintain their current health status, but do not provide sufficient stimuli for obtaining substantial advancements in their health or fitness. This conclusion highlights the irreplaceable value of human expertise and experience in the realm of exercise prescription. Live fitness specialists, with their ability to dynamically adjust and personalise workout plans, maintain a decisive advantage over current AI technology in fostering meaningful physiological adaptations and health benefits.

Chatbots AI technology, as it stands (mid 2023), appears to possess limitations that restrict its potential within the realm of personalised exercise prescription. Despite offering generally safe recommendations, AI-generated fitness plans seem to lack the personalisation, creativity, and variability that a fitness specialist can provide. For instance, a fitness specialist could consider the patient’s fluctuating energy levels and tailor the intensity of workouts, accordingly, ensuring that the user remains engaged and motivated while avoiding burnout. This level of holistic approach leads to personalization and creativity beyond what AI-generated plans can offer, as it takes into account unique user attributes and adapts in ways that resonate with their individual preferences and needs. Even with technological advances, the full replacement of human expertise in the exercise prescription field by AI systems seems distant. This study highlights areas of improvement for AI systems, such as enhancing real-time interactions, improving personalisation and adaptability algorithms, and integrating a greater degree of progression in exercise intensity and variability in training. Only through such advancements can AI provide exercise recommendations that come closer to matching the quality and effectiveness of a live fitness specialist.

## Data Availability

The data that support the findings of this study are openly available upon request from the corresponding author.

## Declaration

We want to emphasize that the interpretation of the results in this study is somewhat subjective, as it relies on the authors’ experience with advanced language models. Although we endeavoured to maintain objectivity, it is important to acknowledge that different reviewers may hold varying interpretations of the findings. In an effort to mitigate this limitation, we sought the feedback of a substantial number of highly active experts in sports medicine, exercise sciences, and exercise physiology, as indicated in the author list. Their insights provided a nuanced critical evaluation of chatGPT-4’s capabilities in exercise prescription for individuals living with medical conditions and seeking exercise programs.

In the interest of transparency, it’s essential to clarify our authorship criteria. The listed authors are those who have made significant and sustained contributions to the study. Others, who made valuable but intermittent contributions, are duly recognized in the ‘Acknowledgements’ section. Additionally, while many specialists were approached and expressed initial interest, not all were able to contribute to a degree that warranted formal recognition. Those who neither made significant contributions nor responded within the agreed-upon timeframe have not been acknowledged in the manuscript.

Throughout this endeavor, our primary goal has been to maintain integrity, transparency, and an unwavering commitment to academic rigor.

## Author’s ORCID

Ismail Dergaa: 0000-0001-8091-1856

Helmi Ben Saad: 0000-0002-7477-2965

Abdelfatteh El Omri: 0000-0003-4112-7924

Jordan M. Glenn: 0000-0002-2563-4291

Cain C. T. Clark: 0000-0002-6610-4617

Jad Adrian Washif: 0000-0001-8543-4489

Noomen Guelmami: 0000-0002-4959-9726

Omar Hammouda: 0000-0002-5002-687X

Ramzi A. Al-Horani: 0000-0002-6915-816X

Luis Felipe Reynoso-Sánchez: 0000-0001-9008-5105

Mohamed Romdhani: 0000-0002-1715-1863

Laisa Liane Paineiras-Domingos: 0000-0003-3451-5056

Rodrigo L. Vancini: 0000-0003-1981-1092

Morteza Taheri: 0000-0001-8031-3792

Leonardo Jose Mataruna-Dos-Santos: 0000-0001-9456-5974

Khaled Trabelsi: 0000-0003-2623-9557

Hamdi Chtourou: 0000-0002-5482-9151

Makram Zghibi: 0000-0001-5500-930X

Özgür Eken: 0000-0002-5488-3158

Sarya Swed: 0000-0002-9983-2020

Mohamed Ben Aissa: 0000-0001-7694-1059

Hossam H. Shawki: 0000-0002-1962-9701

Iñigo Mujika: 0000-0002-8143-9132

Stephen Seiler: 0000-0001-8024-5232

Piotr Zmijewski: 0000-0002-5570-9573

David B. Pyne: 0000-0003-1555-5079

Beat Knechtle: 0000-0002-2412-9103

Irfan M Asif: 0000-0003-0655-6264

Jonathan A Drezner: 0000-0003-3519-9120

Øyvind Sandbakk: 0000-0002-9014-5152

Karim Chamari: 0000-0001-9178-7678
